# The Madman

**Published:** 1870-09

**Authors:** 


### THE MADMAN.

More than forty years ago we saw a young man pacing round an enclosure in the bright sunshine of summer; he was well dressed and had the appearance of having been accustomed to society, and of an intellectual, cultivated mind; it had been his occupation some years, it has been his occupation ever since, until the other day he died, having been the inmate of a lunatic asylum for fifty years. His father and mother were possessors of vigorous constitutions, and of unusual mental abilities. They both died approaching four score years.

The maladies of mind and body, their peculiarities, weaknesses, deficiencies, deformities and monstrosities, have their ultimate foundations laid in the condition of the mind and body of the parent during the night-time of life. All physiological facts in this connection show that during the night, the powers of life, the vitality of man, is at its lowest point. Every one knows that the more healthful the parents are, the more healthful the children will be; this is the general rule.

In the case above mentioned, an easy explanation is found in the fact, that in his youth the father was a gambler, spending a large portion of the night in card-playing and drinking with a near relative of ours; coming home many times with a mind almost exhausted by the excitement of a night's play, and the body under the influence of long hours of whiskey carousals. No wonder that the son of his youth should have been

FIFTY YEARS A MANIAC!

Our book on Sleep, or the Hygiene of the Night, discusses this and kindred subjects in all their bearings. As far as we know of the millions of books published, no one has ever come from the press, the exclusive object of which was to show the importance of a healthful condition of mind and body at night, and the bearing these have on the mental and physical condition of individuals and communities. The following is the preface of the book: —

"Between the closing of the chamber door at night and its opening in the morning, a third of human life passes away, and upon the manner of its employment the physical, mental and moral character of man largely depends.

"If so considerable a portion of existence is spent in breathing an impure air, bodily disease and a premature death will inevitably result; to this subject the first part of the book is devoted.

"If during this third of life improper habits are cultivated, or there is an intemperate indulgence in some of the natural propensities, moral contamination and physical deterioration follow.

"If by the above or other causes a full proportion of sound and regular sleep is prevented, the mind sooner or later fails of its elasticity, its vigor, and its life, to be followed by nervousness, weakness of intellect, softening of the brain, insanity, and death. Hence it is intended to urge the practical and individual application of these truths on the part of husbands, wives, and children; hence every intelligent reader has a personal interest in ascertaining the most healthful method of passing a portion of his existence which has such important bearings, — of acquainting himself with

"THE HYGIENE OF THE NIGHT."

